# 5-Chloro-2-methyl-3-phenyl­sulfonyl-1-benzofuran

**DOI:** 10.1107/S1600536808015699

**Published:** 2008-06-07

**Authors:** Hong Dae Choi, Pil Ja Seo, Byeng Wha Son, Uk Lee

**Affiliations:** a Department of Chemistry, Dongeui University, San 24 Kaya-dong, Busanjin-gu, Busan 614-714, Republic of Korea; b Department of Chemistry, Pukyong National University, 599-1 Daeyeon 3-dong, Nam-gu, Busan 608-737, Republic of Korea

## Abstract

The title compound, C_15_H_11_ClO_3_S, was prepared by the oxidation of 5-chloro-2-methyl-3-phenyl­sulfanyl-1-benzofuran with 3-chloro­peroxy­benzoic acid. There are two symmetry-independent mol­ecules in the asymmetric unit. The dihedral angles formed by the phenyl ring and the plane of the benzofuran system are 77.80 (8) and 78.34 (8)°. The crystal structure is stabilized by aromatic π–π stacking inter­actions between the furan ring and the benzene rings of neighbouring benzofuran fragments from two symmetry-independent mol­ecules; the centroid–centroid distances within the stacks are 3.689 (4), 3.702 (4), 3.825 (4) and 3.826 (4) Å. Additionally, the stacked mol­ecules exhibit inter- and intra­molecular C—H⋯O inter­actions.

## Related literature

For the crystal structures of similar 2-methyl-3-phenyl­sulfonyl-1-benzofuran derivatives, see: Choi *et al.* (2008*a* ▶,*b* ▶).
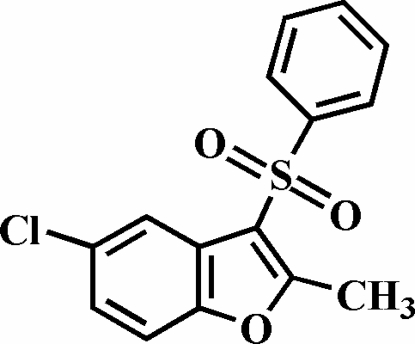


## Experimental

### 

#### Crystal data

C_15_H_11_ClO_3_S
*M* *_r_* = 306.76
Triclinic, 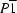

*a* = 7.4029 (7) Å
*b* = 9.2669 (9) Å
*c* = 20.889 (2) Å
α = 100.953 (2)°
β = 95.626 (2)°
γ = 104.212 (2)°
*V* = 1348.0 (2) Å^3^
*Z* = 4
Mo *K*α radiation
μ = 0.44 mm^−1^
*T* = 173 (2) K
0.50 × 0.50 × 0.30 mm

#### Data collection

Bruker SMART CCD diffractometer
Absorption correction: multi-scan (*SADABS*; Sheldrick, 2000 ▶) *T*_min_ = 0.795, *T*_max_ = 0.870
9232 measured reflections
4555 independent reflections
4119 reflections with *I* > 2σ(*I*)
*R*_int_ = 0.036

#### Refinement

*R*[*F* ^2^ > 2σ(*F* ^2^)] = 0.045
*wR*(*F* ^2^) = 0.116
*S* = 1.16
4555 reflections
363 parameters
H-atom parameters constrained
Δρ_max_ = 0.36 e Å^−3^
Δρ_min_ = −0.43 e Å^−3^

### 

Data collection: *SMART* (Bruker, 2001 ▶); cell refinement: *SAINT* (Bruker, 2001 ▶); data reduction: *SAINT*; program(s) used to solve structure: *SHELXS97* (Sheldrick, 2008 ▶); program(s) used to refine structure: *SHELXL97* (Sheldrick, 2008 ▶); molecular graphics: *ORTEP-3* (Farrugia, 1997 ▶) and *DIAMOND* (Brandenburg, 1998 ▶); software used to prepare material for publication: *SHELXL97*.

## Supplementary Material

Crystal structure: contains datablocks global, I. DOI: 10.1107/S1600536808015699/rk2091sup1.cif

Structure factors: contains datablocks I. DOI: 10.1107/S1600536808015699/rk2091Isup2.hkl

Additional supplementary materials:  crystallographic information; 3D view; checkCIF report

## Figures and Tables

**Table 1:** Hydrogen-bond geometry (Å, °)

*D*—H⋯*A*	*D*—H	H⋯*A*	*D*⋯*A*	*D*—H⋯*A*
C15—H15*A*⋯O2	0.98	2.44	3.153 (4)	130
C14—H14⋯O3^i^	0.95	2.51	3.429 (4)	164
C29—H29⋯O5^ii^	0.95	2.51	3.453 (4)	170
C30—H30*A*⋯O6	0.98	2.42	3.141 (4)	130

Symmetry codes: (i) 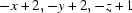; (ii) 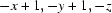.

**Fig. 1.**
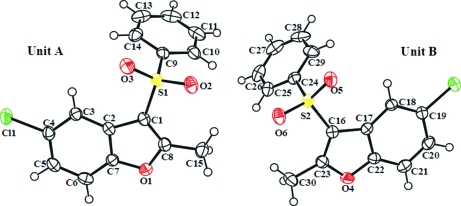
The molecular structure of the title compound with the atom numbering scheme. The displacement ellipsoids are drawn at the 50% probability level. H atoms are presented as a small spheres of arbitrary radius.

**Fig. 2.**
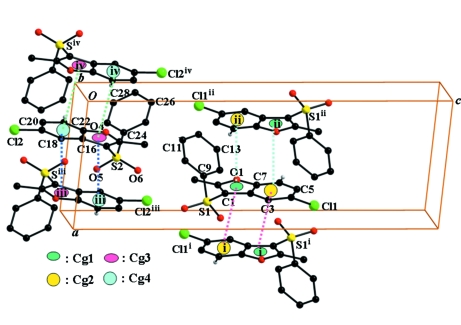
The π–π interactions (dotted lines) in the title compound. Cg denotes the ring centroids. Symmetry codes: (i) -x+2, -y+1, -z+1; (ii) -x+1, -y+1, -z+1; (iii) -x+1, -y, -z; (iv) -x, -y, -z.

**Fig. 3.**
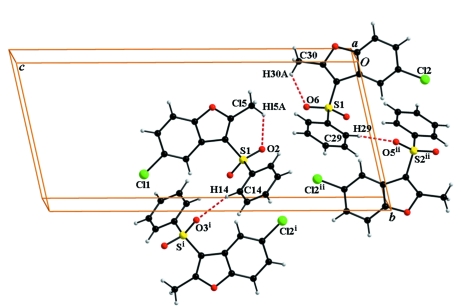
C—H···O interactions (dotted lines) in the title compound. Symmetry codes: (i) -x+2, -y+2, -z+1; (ii) -x+1, -y+1, -z.

## supplementary crystallographic information

### Comment

This work is related to our communications on the synthesis and structure of 2-methyl-3-phenylsulfonyl-1-benzofuran analogues, *viz*. 5-bromo-2-methyl-3-phenylsulfonyl-1-benzofuran (Choi *et al.*, 2008*a*) and 2,5-dimethyl-3-phenylsulfonyl-1-benzofuran (Choi *et al.*, 2008*b*). Herein we report the crystal and molecular structure of the title compound, 5-chloro-2-methyl-3-phenylsulfonyl-1-benzofuran C_15_H_11_ClO_3_S, (I), (Fig. 1).

The benzofuran unit is essentially planar, with a max deviation of 0.015 (2) Å for unit A, and 0.020 (2) Å for unit B, respectively, from the least-squares plane defined by the nine constituent atoms. In the title compound, the dihedral angles formed by the benzofuran fragment and the plane of the phenyl ring are 77.80 (8)° in unit A and 78.34 (8)° in unit B, respectively. The crystal packing (Fig. 2) is stabilized by four different π–π interactions within each stack of molecules; one between the furan ring (*Cg*1) and an adjacent benzene ring (*Cg*2^i^) [distance 3.702 (4) Å], a second between the furan ring (*Cg*1) and an adjacent benzene ring (*Cg*2^ii^) [distance 3.826 (4) Å], a third between the furan ring (*Cg*3) and an adjacent benzene ring (*Cg*4^iii^) [distance 3.689 (4) Å], a fourth between the furan ring (*Cg*3) and an adjacent benzene ring (*Cg*4^iv^) [distance 3.825 (4) Å], (*Cg*1, *Cg*2, *Cg*3, and *Cg*4 are the centroids of the O1/C8/C1/C2/C7 furan ring, the C2/C3/C4/C5/C6/C7 benzene ring, the O4/C23/C16/C17/C22 furan ring, the C17/C18/C19/C20/C21/C22 benzene ring, respectively, symmetry code as in Fig. 2). The molecular packing is further stabilized by inter- and intramolecular C—H···O hydrogen bonds (Fig. 3 and Table; symmetry codes as in Fig. 3).

### Experimental

77% 3-chloroperoxybenzoic acid (471 mg, 2.1 mmol) was added in small portions to a stirred solution of 5-chloro-2-methyl-3-phenylsulfanyl-1-benzofuran (275 mg, 1.0 mmol) in dichloromethane (30 ml) at 273 K. After being stirred for 4 h at room temperature, the mixture was washed with saturated sodium bicarbonate solution and the organic layer was separated, dried over magnesium sulfate, filtered and concentrated in vacuum. The residue was purified by column chromatography (hexane–ethylacetate, 2:1 *v*/*v*) to afford the I as a colorless solid [yield 81%, m.p. 468–469 K; *R*_f_ = 0.61 (hexane–ethylacetate, 2:1 *v*/*v*)]. Single crystals suitable for X-ray diffraction were prepared by evaporation of a solution of the I in chloroform at room temperature. Spectroscopic analysis: ^1^H NMR (CDCl_3_, 400 MHz) δ 2.80 (s, 3H), 7.28 (d, J = 2.16 Hz, 1H), 7.33 (s, 1*H*), 7.51–7.55 (m, 2H), 7.58–7.61 (m, 1H), 7.88 (d, J = 2.20 Hz, 1H), 7.98–8.04 (m, 2H); EI-MS 308 [*M*+2], 306 [*M*^+^].

### Refinement

All H atoms were geometrically positioned and refined using a riding model, with C—H = 0.95 Å for aromatic H atoms and 0.98 Å for methyl H atoms, respectively, and with *U*_iso_(H) = 1.2*U*_eq_(C) for aromatic and *U*_iso_(H) = 1.5*U*_eq_(C) for methyl H atoms.

### Crystal data

**Table:**

C_15_H_11_ClO_3_S	*Z* = 4
*M**_r_* = 306.76	*F*_000_ = 632
Triclinic, *P*1	*D*_x_ = 1.512 Mg m^−^^3^
Hall symbol: -P 1	Melting point = 468–469 K
*a* = 7.4029 (7) Å	Mo *K*α radiation λ = 0.71073 Å
*b* = 9.2669 (9) Å	Cell parameters from 7490 reflections
*c* = 20.889 (2) Å	θ = 2.3–28.2º
α = 100.953 (2)º	µ = 0.44 mm^−^^1^
β = 95.626 (2)º	*T* = 173 (2) K
γ = 104.212 (2)º	Block, colourless
*V* = 1348.0 (2) Å^3^	0.50 × 0.50 × 0.30 mm

### Data collection

**Table:**

Bruker SMART CCD diffractometer	4555 independent reflections
Radiation source: fine-focus sealed tube	4119 reflections with *I* > 2σ(*I*)
Monochromator: graphite	*R*_int_ = 0.036
Detector resolution: 10.0 pixels mm^-1^	θ_max_ = 25.0º
*T* = 173(2) K	θ_min_ = 1.0º
φ and ω scans	*h* = −8→8
Absorption correction: multi-scan(SADABS; Sheldrick, 2000)	*k* = −11→11
*T*_min_ = 0.795, *T*_max_ = 0.870	*l* = −24→24
9232 measured reflections	

### Refinement

**Table:**

Refinement on *F*^2^	Secondary atom site location: difference Fourier map
Least-squares matrix: full	Hydrogen site location: inferred from neighbouring sites
*R*[*F*^2^ > 2σ(*F*^2^)] = 0.045	H-atom parameters constrained
*wR*(*F*^2^) = 0.116	*w* = 1/[σ^2^(*F*_o_^2^) + (0.0289*P*)^2^ + 1.6847*P*] where *P* = (*F*_o_^2^ + 2*F*_c_^2^)/3
*S* = 1.16	(Δ/σ)_max_ < 0.001
4555 reflections	Δρ_max_ = 0.36 e Å^−^^3^
363 parameters	Δρ_min_ = −0.43 e Å^−^^3^
Primary atom site location: structure-invariant direct methods	Extinction correction: none

### Special details

**Table:**

Geometry. All s.u.'s (except the s.u. in the dihedral angle between two l.s. planes) are estimated using the full covariance matrix. The cell s.u.'s are taken into account individually in the estimation of s.u.'s in distances, angles and torsion angles; correlations between s.u.'s in cell parameters are only used when they are defined by crystal symmetry. An approximate (isotropic) treatment of cell s.u.'s is used for estimating s.u.'s involving l.s. planes.
Refinement. Refinement of *F*^2^ against ALL reflections. The weighted *R*-factor *wR* and goodness of fit *S* are based on *F*^2^, conventional *R*-factors *R* are based on *F*, with *F* set to zero for negative *F*^2^. The threshold expression of *F*^2^ >σ(*F*^2^) is used only for calculating *R*-factors(gt) *etc*. and is not relevant to the choice of reflections for refinement. *R*-factors based on *F*^2^ are statistically about twice as large as those based on *F*, and *R*-factors based on ALL data will be even larger.

### Fractional atomic coordinates and isotropic or equivalent isotropic displacement parameters (Å^2^)

**Table:**

	*x*	*y*	*z*	*U*_iso_*/*U*_eq_	
Cl1	0.94027 (12)	0.83729 (9)	0.68782 (4)	0.0390 (2)	
S1	0.89392 (10)	0.73758 (8)	0.38915 (3)	0.02487 (18)	
O1	0.6676 (3)	0.3633 (2)	0.44767 (10)	0.0285 (4)	
O2	0.9367 (3)	0.6705 (3)	0.32655 (10)	0.0344 (5)	
O3	1.0381 (3)	0.8528 (2)	0.43567 (10)	0.0327 (5)	
C1	0.8078 (4)	0.5920 (3)	0.42946 (13)	0.0235 (6)	
C2	0.8110 (4)	0.6091 (3)	0.50012 (13)	0.0219 (6)	
C3	0.8793 (4)	0.7294 (3)	0.55589 (13)	0.0242 (6)	
H3	0.9389	0.8302	0.5524	0.029*	
C4	0.8551 (4)	0.6929 (3)	0.61626 (14)	0.0265 (6)	
C5	0.7692 (4)	0.5459 (4)	0.62322 (14)	0.0297 (6)	
H5	0.7575	0.5271	0.6660	0.036*	
C6	0.7012 (4)	0.4275 (3)	0.56827 (15)	0.0303 (7)	
H6	0.6421	0.3266	0.5718	0.036*	
C7	0.7242 (4)	0.4643 (3)	0.50790 (14)	0.0242 (6)	
C8	0.7201 (4)	0.4438 (3)	0.40081 (14)	0.0262 (6)	
C9	0.7018 (4)	0.8137 (3)	0.37439 (14)	0.0246 (6)	
C10	0.5757 (4)	0.7514 (4)	0.31622 (15)	0.0311 (7)	
H10	0.5941	0.6705	0.2843	0.037*	
C11	0.4221 (5)	0.8097 (4)	0.30559 (18)	0.0406 (8)	
H11	0.3351	0.7694	0.2659	0.049*	
C12	0.3958 (5)	0.9256 (4)	0.3524 (2)	0.0445 (9)	
H12	0.2897	0.9641	0.3449	0.053*	
C13	0.5210 (5)	0.9865 (4)	0.40995 (19)	0.0421 (8)	
H13	0.5015	1.0672	0.4417	0.051*	
C14	0.6760 (4)	0.9309 (3)	0.42202 (16)	0.0316 (7)	
H14	0.7624	0.9719	0.4619	0.038*	
C15	0.6662 (5)	0.3533 (4)	0.33182 (15)	0.0352 (7)	
H15A	0.7152	0.4174	0.3017	0.053*	
H15B	0.5285	0.3172	0.3212	0.053*	
H15C	0.7193	0.2655	0.3270	0.053*	
Cl2	0.24348 (13)	0.14348 (9)	−0.18646 (4)	0.0400 (2)	
S2	0.49179 (10)	0.35480 (8)	0.11049 (3)	0.02486 (18)	
O4	0.2303 (3)	−0.0834 (2)	0.05447 (10)	0.0303 (5)	
O5	0.5910 (3)	0.4231 (2)	0.06317 (10)	0.0315 (5)	
O6	0.5927 (3)	0.3559 (3)	0.17302 (10)	0.0339 (5)	
C16	0.3752 (4)	0.1665 (3)	0.07152 (14)	0.0236 (6)	
C17	0.3089 (4)	0.1109 (3)	0.00122 (13)	0.0225 (6)	
C18	0.3154 (4)	0.1733 (3)	−0.05452 (13)	0.0241 (6)	
H18	0.3716	0.2787	−0.0515	0.029*	
C19	0.2356 (4)	0.0736 (3)	−0.11451 (14)	0.0274 (6)	
C20	0.1522 (4)	−0.0821 (3)	−0.12108 (15)	0.0309 (7)	
H20	0.1012	−0.1456	−0.1635	0.037*	
C21	0.1440 (4)	−0.1436 (3)	−0.06597 (15)	0.0315 (7)	
H21	0.0877	−0.2489	−0.0690	0.038*	
C22	0.2222 (4)	−0.0437 (3)	−0.00587 (14)	0.0244 (6)	
C23	0.3230 (4)	0.0464 (3)	0.10065 (14)	0.0276 (6)	
C24	0.3087 (4)	0.4413 (3)	0.12603 (14)	0.0254 (6)	
C25	0.2273 (4)	0.4267 (3)	0.18244 (15)	0.0317 (7)	
H25	0.2729	0.3742	0.2126	0.038*	
C26	0.0798 (5)	0.4891 (4)	0.19426 (17)	0.0408 (8)	
H26	0.0228	0.4797	0.2326	0.049*	
C27	0.0152 (5)	0.5653 (4)	0.15001 (19)	0.0447 (9)	
H27	−0.0866	0.6083	0.1582	0.054*	
C28	0.0965 (5)	0.5797 (4)	0.0943 (2)	0.0442 (9)	
H28	0.0508	0.6328	0.0645	0.053*	
C29	0.2448 (4)	0.5171 (3)	0.08137 (17)	0.0348 (7)	
H29	0.3010	0.5262	0.0428	0.042*	
C30	0.3420 (5)	0.0266 (4)	0.16940 (16)	0.0396 (8)	
H30A	0.4237	0.1205	0.1984	0.059*	
H30B	0.3975	−0.0583	0.1720	0.059*	
H30C	0.2173	0.0044	0.1833	0.059*	

### Atomic displacement parameters (Å^2^)

**Table:**

	*U*^11^	*U*^22^	*U*^33^	*U*^12^	*U*^13^	*U*^23^
Cl1	0.0524 (5)	0.0418 (5)	0.0215 (4)	0.0176 (4)	0.0002 (3)	−0.0001 (3)
S1	0.0231 (4)	0.0292 (4)	0.0215 (4)	0.0035 (3)	0.0058 (3)	0.0071 (3)
O1	0.0314 (11)	0.0232 (10)	0.0290 (11)	0.0051 (9)	0.0025 (9)	0.0052 (8)
O2	0.0362 (12)	0.0446 (13)	0.0254 (11)	0.0126 (10)	0.0133 (9)	0.0088 (9)
O3	0.0260 (11)	0.0348 (12)	0.0316 (12)	−0.0031 (9)	0.0010 (9)	0.0100 (9)
C1	0.0207 (14)	0.0289 (15)	0.0217 (14)	0.0066 (11)	0.0044 (11)	0.0070 (11)
C2	0.0173 (13)	0.0282 (14)	0.0217 (14)	0.0063 (11)	0.0039 (11)	0.0084 (11)
C3	0.0222 (14)	0.0257 (14)	0.0239 (14)	0.0057 (11)	0.0023 (11)	0.0054 (11)
C4	0.0273 (15)	0.0319 (16)	0.0218 (14)	0.0123 (12)	0.0035 (12)	0.0040 (12)
C5	0.0320 (16)	0.0381 (17)	0.0239 (15)	0.0135 (13)	0.0068 (13)	0.0125 (13)
C6	0.0304 (16)	0.0303 (16)	0.0351 (17)	0.0089 (13)	0.0093 (13)	0.0160 (13)
C7	0.0221 (14)	0.0243 (14)	0.0259 (15)	0.0074 (11)	0.0003 (11)	0.0051 (11)
C8	0.0241 (14)	0.0311 (15)	0.0242 (15)	0.0091 (12)	0.0036 (12)	0.0062 (12)
C9	0.0252 (14)	0.0237 (14)	0.0234 (14)	0.0007 (11)	0.0057 (12)	0.0085 (11)
C10	0.0324 (16)	0.0324 (16)	0.0271 (16)	0.0032 (13)	0.0025 (13)	0.0116 (13)
C11	0.0331 (17)	0.0439 (19)	0.046 (2)	0.0026 (15)	−0.0019 (15)	0.0262 (16)
C12	0.0329 (18)	0.0394 (19)	0.073 (3)	0.0131 (15)	0.0128 (18)	0.0329 (19)
C13	0.043 (2)	0.0264 (16)	0.062 (2)	0.0106 (15)	0.0213 (18)	0.0140 (16)
C14	0.0316 (16)	0.0244 (15)	0.0336 (17)	−0.0012 (12)	0.0078 (13)	0.0039 (12)
C15	0.0387 (18)	0.0331 (17)	0.0288 (16)	0.0081 (14)	−0.0004 (14)	−0.0004 (13)
Cl2	0.0602 (5)	0.0376 (4)	0.0227 (4)	0.0147 (4)	0.0028 (4)	0.0079 (3)
S2	0.0220 (4)	0.0284 (4)	0.0208 (4)	0.0028 (3)	0.0013 (3)	0.0037 (3)
O4	0.0357 (12)	0.0243 (10)	0.0332 (11)	0.0074 (9)	0.0082 (9)	0.0119 (9)
O5	0.0284 (11)	0.0342 (12)	0.0263 (11)	−0.0016 (9)	0.0059 (9)	0.0053 (9)
O6	0.0291 (11)	0.0441 (13)	0.0258 (11)	0.0092 (10)	−0.0025 (9)	0.0057 (9)
C16	0.0195 (14)	0.0266 (14)	0.0247 (14)	0.0060 (11)	0.0040 (11)	0.0054 (11)
C17	0.0191 (13)	0.0224 (14)	0.0255 (14)	0.0060 (11)	0.0042 (11)	0.0033 (11)
C18	0.0246 (14)	0.0217 (14)	0.0245 (14)	0.0040 (11)	0.0029 (12)	0.0051 (11)
C19	0.0316 (16)	0.0287 (15)	0.0229 (15)	0.0099 (12)	0.0046 (12)	0.0057 (12)
C20	0.0308 (16)	0.0271 (15)	0.0286 (16)	0.0059 (13)	−0.0029 (13)	−0.0023 (12)
C21	0.0296 (16)	0.0244 (15)	0.0378 (17)	0.0057 (12)	0.0035 (13)	0.0031 (13)
C22	0.0233 (14)	0.0239 (14)	0.0278 (15)	0.0075 (11)	0.0060 (12)	0.0074 (12)
C23	0.0282 (15)	0.0306 (16)	0.0282 (15)	0.0125 (12)	0.0084 (12)	0.0085 (12)
C24	0.0204 (14)	0.0215 (14)	0.0290 (15)	0.0004 (11)	−0.0008 (12)	0.0018 (11)
C25	0.0331 (16)	0.0329 (16)	0.0251 (15)	0.0065 (13)	0.0018 (13)	0.0011 (12)
C26	0.0364 (18)	0.0406 (19)	0.0387 (19)	0.0082 (15)	0.0091 (15)	−0.0068 (15)
C27	0.0289 (17)	0.0283 (17)	0.068 (3)	0.0069 (14)	0.0010 (17)	−0.0062 (16)
C28	0.0361 (18)	0.0215 (16)	0.072 (3)	0.0024 (14)	−0.0053 (18)	0.0160 (16)
C29	0.0304 (16)	0.0282 (16)	0.0431 (19)	−0.0012 (13)	0.0009 (14)	0.0155 (14)
C30	0.054 (2)	0.0414 (19)	0.0314 (17)	0.0169 (16)	0.0119 (15)	0.0183 (14)

### Geometric parameters (Å, °)

**Table:**

Cl1—C4	1.750 (3)	Cl2—C19	1.746 (3)
S1—O3	1.437 (2)	S2—O6	1.437 (2)
S1—O2	1.437 (2)	S2—O5	1.438 (2)
S1—C1	1.746 (3)	S2—C16	1.742 (3)
S1—C9	1.762 (3)	S2—C24	1.762 (3)
O1—C8	1.369 (3)	O4—C23	1.368 (4)
O1—C7	1.375 (3)	O4—C22	1.378 (3)
C1—C8	1.357 (4)	C16—C23	1.361 (4)
C1—C2	1.451 (4)	C16—C17	1.452 (4)
C2—C7	1.386 (4)	C17—C22	1.392 (4)
C2—C3	1.404 (4)	C17—C18	1.395 (4)
C3—C4	1.383 (4)	C18—C19	1.386 (4)
C3—H3	0.9500	C18—H18	0.9500
C4—C5	1.396 (4)	C19—C20	1.398 (4)
C5—C6	1.383 (4)	C20—C21	1.378 (4)
C5—H5	0.9500	C20—H20	0.9500
C6—C7	1.383 (4)	C21—C22	1.386 (4)
C6—H6	0.9500	C21—H21	0.9500
C8—C15	1.484 (4)	C23—C30	1.480 (4)
C9—C10	1.388 (4)	C24—C29	1.383 (4)
C9—C14	1.391 (4)	C24—C25	1.389 (4)
C10—C11	1.391 (4)	C25—C26	1.377 (4)
C10—H10	0.9500	C25—H25	0.9500
C11—C12	1.375 (5)	C26—C27	1.382 (5)
C11—H11	0.9500	C26—H26	0.9500
C12—C13	1.374 (5)	C27—C28	1.374 (5)
C12—H12	0.9500	C27—H27	0.9500
C13—C14	1.389 (5)	C28—C29	1.388 (5)
C13—H13	0.9500	C28—H28	0.9500
C14—H14	0.9500	C29—H29	0.9500
C15—H15A	0.9800	C30—H30A	0.9800
C15—H15B	0.9800	C30—H30B	0.9800
C15—H15C	0.9800	C30—H30C	0.9800
			
O3—S1—O2	119.89 (13)	O6—S2—O5	119.93 (13)
O3—S1—C1	106.92 (13)	O6—S2—C16	108.60 (13)
O2—S1—C1	108.61 (13)	O5—S2—C16	107.16 (13)
O3—S1—C9	107.87 (13)	O6—S2—C24	107.79 (13)
O2—S1—C9	108.20 (13)	O5—S2—C24	108.08 (13)
C1—S1—C9	104.28 (13)	C16—S2—C24	104.18 (13)
C8—O1—C7	106.9 (2)	C23—O4—C22	106.9 (2)
C8—C1—C2	107.3 (2)	C23—C16—C17	107.4 (2)
C8—C1—S1	126.7 (2)	C23—C16—S2	127.1 (2)
C2—C1—S1	126.0 (2)	C17—C16—S2	125.5 (2)
C7—C2—C3	119.6 (2)	C22—C17—C18	119.4 (3)
C7—C2—C1	104.7 (2)	C22—C17—C16	104.4 (2)
C3—C2—C1	135.7 (3)	C18—C17—C16	136.2 (3)
C4—C3—C2	116.2 (3)	C19—C18—C17	116.5 (3)
C4—C3—H3	121.9	C19—C18—H18	121.7
C2—C3—H3	121.9	C17—C18—H18	121.7
C3—C4—C5	123.4 (3)	C18—C19—C20	123.5 (3)
C3—C4—Cl1	118.5 (2)	C18—C19—Cl2	118.9 (2)
C5—C4—Cl1	118.1 (2)	C20—C19—Cl2	117.6 (2)
C6—C5—C4	120.4 (3)	C21—C20—C19	120.0 (3)
C6—C5—H5	119.8	C21—C20—H20	120.0
C4—C5—H5	119.8	C19—C20—H20	120.0
C5—C6—C7	116.2 (3)	C20—C21—C22	116.5 (3)
C5—C6—H6	121.9	C20—C21—H21	121.7
C7—C6—H6	121.9	C22—C21—H21	121.7
O1—C7—C6	125.2 (3)	O4—C22—C21	125.3 (3)
O1—C7—C2	110.6 (2)	O4—C22—C17	110.7 (2)
C6—C7—C2	124.2 (3)	C21—C22—C17	124.0 (3)
C1—C8—O1	110.5 (2)	C16—C23—O4	110.6 (2)
C1—C8—C15	134.8 (3)	C16—C23—C30	134.7 (3)
O1—C8—C15	114.7 (3)	O4—C23—C30	114.7 (3)
C10—C9—C14	121.5 (3)	C29—C24—C25	121.4 (3)
C10—C9—S1	119.0 (2)	C29—C24—S2	120.0 (2)
C14—C9—S1	119.5 (2)	C25—C24—S2	118.5 (2)
C9—C10—C11	118.7 (3)	C26—C25—C24	119.4 (3)
C9—C10—H10	120.7	C26—C25—H25	120.3
C11—C10—H10	120.7	C24—C25—H25	120.3
C12—C11—C10	120.2 (3)	C25—C26—C27	119.6 (3)
C12—C11—H11	119.9	C25—C26—H26	120.2
C10—C11—H11	119.9	C27—C26—H26	120.2
C13—C12—C11	120.8 (3)	C28—C27—C26	120.8 (3)
C13—C12—H12	119.6	C28—C27—H27	119.6
C11—C12—H12	119.6	C26—C27—H27	119.6
C12—C13—C14	120.5 (3)	C27—C28—C29	120.5 (3)
C12—C13—H13	119.8	C27—C28—H28	119.7
C14—C13—H13	119.8	C29—C28—H28	119.7
C13—C14—C9	118.4 (3)	C24—C29—C28	118.3 (3)
C13—C14—H14	120.8	C24—C29—H29	120.9
C9—C14—H14	120.8	C28—C29—H29	120.9
C8—C15—H15A	109.5	C23—C30—H30A	109.5
C8—C15—H15B	109.5	C23—C30—H30B	109.5
H15A—C15—H15B	109.5	H30A—C30—H30B	109.5
C8—C15—H15C	109.5	C23—C30—H30C	109.5
H15A—C15—H15C	109.5	H30A—C30—H30C	109.5
H15B—C15—H15C	109.5	H30B—C30—H30C	109.5
			
O3—S1—C1—C8	−156.6 (2)	O6—S2—C16—C23	23.3 (3)
O2—S1—C1—C8	−25.9 (3)	O5—S2—C16—C23	154.3 (2)
C9—S1—C1—C8	89.3 (3)	C24—S2—C16—C23	−91.3 (3)
O3—S1—C1—C2	25.9 (3)	O6—S2—C16—C17	−159.3 (2)
O2—S1—C1—C2	156.6 (2)	O5—S2—C16—C17	−28.4 (3)
C9—S1—C1—C2	−88.2 (3)	C24—S2—C16—C17	86.0 (2)
C8—C1—C2—C7	0.8 (3)	C23—C16—C17—C22	−0.9 (3)
S1—C1—C2—C7	178.7 (2)	S2—C16—C17—C22	−178.7 (2)
C8—C1—C2—C3	179.8 (3)	C23—C16—C17—C18	179.9 (3)
S1—C1—C2—C3	−2.3 (5)	S2—C16—C17—C18	2.1 (5)
C7—C2—C3—C4	0.4 (4)	C22—C17—C18—C19	−1.0 (4)
C1—C2—C3—C4	−178.5 (3)	C16—C17—C18—C19	178.1 (3)
C2—C3—C4—C5	0.4 (4)	C17—C18—C19—C20	−0.2 (4)
C2—C3—C4—Cl1	179.1 (2)	C17—C18—C19—Cl2	−178.6 (2)
C3—C4—C5—C6	−0.7 (4)	C18—C19—C20—C21	0.9 (5)
Cl1—C4—C5—C6	−179.4 (2)	Cl2—C19—C20—C21	179.3 (2)
C4—C5—C6—C7	0.1 (4)	C19—C20—C21—C22	−0.3 (4)
C8—O1—C7—C6	−178.5 (3)	C23—O4—C22—C21	178.1 (3)
C8—O1—C7—C2	0.5 (3)	C23—O4—C22—C17	−0.2 (3)
C5—C6—C7—O1	179.6 (3)	C20—C21—C22—O4	−179.1 (3)
C5—C6—C7—C2	0.7 (4)	C20—C21—C22—C17	−1.0 (4)
C3—C2—C7—O1	−180.0 (2)	C18—C17—C22—O4	180.0 (2)
C1—C2—C7—O1	−0.8 (3)	C16—C17—C22—O4	0.6 (3)
C3—C2—C7—C6	−1.0 (4)	C18—C17—C22—C21	1.7 (4)
C1—C2—C7—C6	178.2 (3)	C16—C17—C22—C21	−177.7 (3)
C2—C1—C8—O1	−0.5 (3)	C17—C16—C23—O4	0.8 (3)
S1—C1—C8—O1	−178.42 (19)	S2—C16—C23—O4	178.58 (19)
C2—C1—C8—C15	177.8 (3)	C17—C16—C23—C30	−178.2 (3)
S1—C1—C8—C15	−0.1 (5)	S2—C16—C23—C30	−0.4 (5)
C7—O1—C8—C1	0.0 (3)	C22—O4—C23—C16	−0.4 (3)
C7—O1—C8—C15	−178.6 (2)	C22—O4—C23—C30	178.8 (2)
O3—S1—C9—C10	157.2 (2)	O6—S2—C24—C29	150.9 (2)
O2—S1—C9—C10	26.1 (3)	O5—S2—C24—C29	19.9 (3)
C1—S1—C9—C10	−89.4 (2)	C16—S2—C24—C29	−93.8 (3)
O3—S1—C9—C14	−25.0 (3)	O6—S2—C24—C25	−31.2 (3)
O2—S1—C9—C14	−156.0 (2)	O5—S2—C24—C25	−162.2 (2)
C1—S1—C9—C14	88.5 (2)	C16—S2—C24—C25	84.0 (2)
C14—C9—C10—C11	0.8 (4)	C29—C24—C25—C26	0.0 (4)
S1—C9—C10—C11	178.6 (2)	S2—C24—C25—C26	−177.8 (2)
C9—C10—C11—C12	−0.7 (4)	C24—C25—C26—C27	−0.1 (5)
C10—C11—C12—C13	0.6 (5)	C25—C26—C27—C28	0.0 (5)
C11—C12—C13—C14	−0.5 (5)	C26—C27—C28—C29	0.3 (5)
C12—C13—C14—C9	0.6 (5)	C25—C24—C29—C28	0.3 (4)
C10—C9—C14—C13	−0.7 (4)	S2—C24—C29—C28	178.0 (2)
S1—C9—C14—C13	−178.5 (2)	C27—C28—C29—C24	−0.4 (5)

### Hydrogen-bond geometry (Å, °)

**Table:**

*D*—H···*A*	*D*—H	H···*A*	*D*···*A*	*D*—H···*A*
C15—H15A···O2	0.98	2.44	3.153 (4)	130
C14—H14···O3^i^	0.95	2.51	3.429 (4)	164
C29—H29···O5^ii^	0.95	2.51	3.453 (4)	170
C30—H30A···O6	0.98	2.42	3.141 (4)	130

Symmetry codes: (i) −*x*+2, −*y*+2, −*z*+1; (ii) −*x*+1, −*y*+1, −*z*.
